# Assessment of Land Cover Status and Change in the World and “the Belt and Road” Region from 2016 to 2020

**DOI:** 10.3390/s23167158

**Published:** 2023-08-14

**Authors:** Aixia Yang, Bo Zhong, Longfei Hu, Ao Kai, Li Li, Fei Zhao, Junjun Wu

**Affiliations:** 1 State Key Laboratory of Remote Sensing Science, Aerospace Information Research Institute, Chinese Academy of Sciences, Beijing 100101, China; yangax@radi.ac.cn (A.Y.); hulf@aircas.ac.cn (L.H.); aokai@aircas.ac.cn (A.K.); lilifs@aircas.ac.cn (L.L.); wujj@radi.ac.cn (J.W.); 2 China Satellite Communications Co., Ltd., Beijing 100190, China; zhaofei@chinasatcom.com

**Keywords:** global, the B&R, land cover, change, transformation characteristics

## Abstract

The assessment of land cover and changes will help to understand the temporal and spatial pattern of land cover in the world and the Belt and Road (B&R) region, and provide reference information for global sustainable development and the Belt and Road construction. In this paper, the 1 km global land cover classification maps of 2016 and 2020 with a high accuracy of 88% are mapped using the Moderate Resolution Imaging Spectroradiometer (MODIS) time series surface reflectance products. Based on the maps, the land cover status of the world and the Belt and Road region, the land cover change from 2016 to 2020, and the mutual transformation characteristics between various types, are analyzed. The research results indicate that from 2016 to 2020, the global change rates of cropland, forest, grassland, and impervious surface are 0.25%, 0.22%, 0.08% and 3.41%, respectively. In the Belt and Road region, the change rates of cropland, forest, grassland, and impervious surface are 0.42%, 0.60%, −0.55% and 2.98% respectively. The assessment results will help to clarify the spatial pattern of land cover change in the five years from 2016 to 2020, so as to provide valuable scientific information for the global realization of sustainable development goals and the construction of the B&R.

## 1. Introduction

With the continuous expansion of the world population and rapid economic growth, the overexploitation of natural resources, shortage of energy and water resources, loss of biodiversity, and environmental and climate issues have become prominent worldwide [1,2]. As a result, the international community has begun to reflect and explore the path of coordinated development between society, economy, and environment. At the United Nations Sustainable Development Summit held in September 2015, nearly 200 countries around the world jointly signed the “2030 Agenda for Sustainable Development”. This summit proposed 17 sustainable development goals (SDGs) and planned a blueprint for the development of human society for the next 15 years, pointing the way for the development and international cooperation of countries around the world [3,4,5].

The Belt and Road (hereinafter referred to as ‘B&R’) Initiative is the abbreviation of the “Silk Road Economic Belt” and the “21st Century Maritime Silk Road”. It aims to establish and strengthen connectivity partnerships among countries along the Belt and Road, build a comprehensive, multi-level, and composite connectivity network, and achieve diversified, independent, balanced, and sustainable development among countries along the Belt and Road [6,7]. As an interconnected community with a shared future, the B&R region is also facing a series of ecological and environmental problems, such as air pollution, water pollution, climate warming, land degradation, and biodiversity reduction, caused by land cover changes caused by human activities [8].

Land cover change is the result of human activities and natural evolution, which can most intuitively reflect the changes in the spatiotemporal pattern of elements in the Earth’s surface system such as the surface energy balance, carbon cycle, water cycle, and biodiversity [9,10,11,12], and is widely used in many fields such as global climate change, earth system modeling, natural resource management and monitoring, food security, and protection [13,14,15,16]. The assessment of land cover and changes will help to understand the temporal and spatial pattern of land cover in the world and the B&R region, and provide reference information for global sustainable development and the Belt and Road construction.

There are many studies on spatiotemporal analysis of land cover/use for the B&R and the world, but most of them use existing classification products. Due to data limitations, high-resolution land cover/use products cannot form long-term continuous observations, so low-resolution land cover/use products are normally used in the analysis of large regional scales. Currently, the longest-lasting and freely available classified product is the Terra and Aqua combined Moderate Resolution Imaging Spectroradiometer (MODIS) Land Cover Type (MCD12Q1). This product provides global land cover types at yearly intervals from 2001 (currently updated to 2020). The product is derived using supervised classifications of MODIS Terra and Aqua reflectance data. Land cover types are derived from the International Geosphere-Biosphere Programme (IGBP), University of Maryland (UMD), Leaf Area Index (LAI), BIOME-Biogeochemical Cycles (BGC), and Plant Functional Types (PFT) classification schemes. The supervised classifications then undergo additional post-processing that incorporates prior knowledge and ancillary information to further refine specific classes. However, the classification accuracy of MCD12Q1 is only 75%, which is even lower in China due to its complex terrain [17]. Therefore, low-resolution land cover products with good continuous consistency and high accuracy are still needed.

In order to make explicit the B&R and global land cover status and change from 2016 to 2020 using land cover products with good continuous consistency and high accuracy, this paper produced high-precision 1 km global land cover maps of 2016 and 2020 based on the MODIS time series surface reflectance, and analyzed the land cover status, change, and mutual transformation characteristics between various types from 2016 to 2020.

## 2. Materials and Methods

### 2.1. Study Area

**Figure 1:**
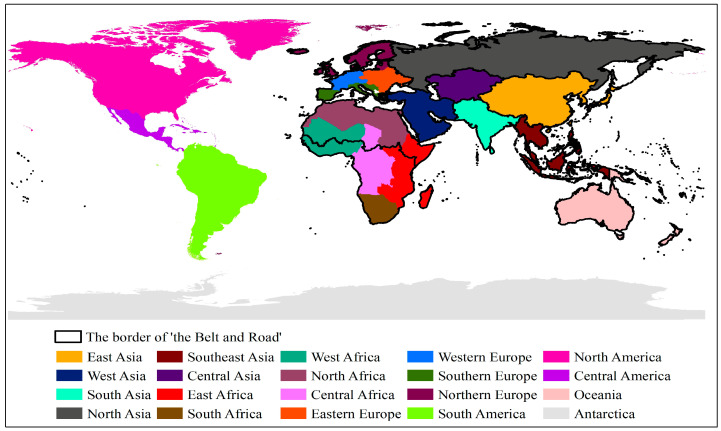
Twenty sub-regions worldwide.

In order to better analyze the global land cover, this paper divides the world into 20 sub-regions based on geographical location (Figure 1). Asia is divided into 6 sub-regions: East Asia, West Asia, South Asia, Southeast Asia, Central Asia, and North Asia. Europe is divided into 4 sub-regions: Southern Europe, Nordic Europe, Eastern Europe, and Western Europe. America is divided into 3 sub-regions: North America, Central America, and South America. Africa is divided into 5 sub-regions: North Africa, East Africa, South Africa, Central Africa, and West Africa. Oceania and Antarctica are the last 2 sub-regions. The B&R is an open international regional economic cooperation network, with the main body running through Asia, Africa and Europe, and no precise spatial scope. The study area of the B&R in this paper refers to the scope of countries and regions that have signed the B&R Initiative cooperation agreement, shown in Figure 1.

### 2.2. Mapping Global Land Cover Classification

**Figure 2:**
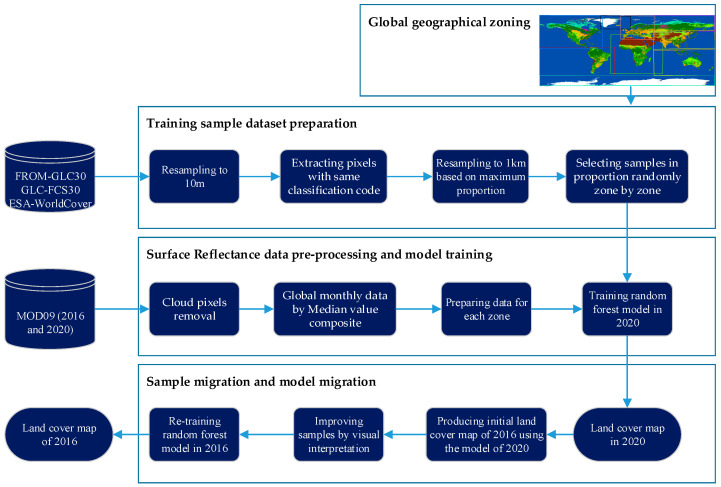
Algorithm flowchart of mapping global land cover classification.

In this paper, the land cover classification maps in 2016 and 2020 are based on MODIS time series surface reflectance products and produced using the technology of geographical zoning and sample migration. The algorithm flow is shown in Figure 2.

The process mainly includes four parts: global geographic zoning, sample dataset production, classification dataset and model training, sample migration, and model migration.

**Figure 3:**
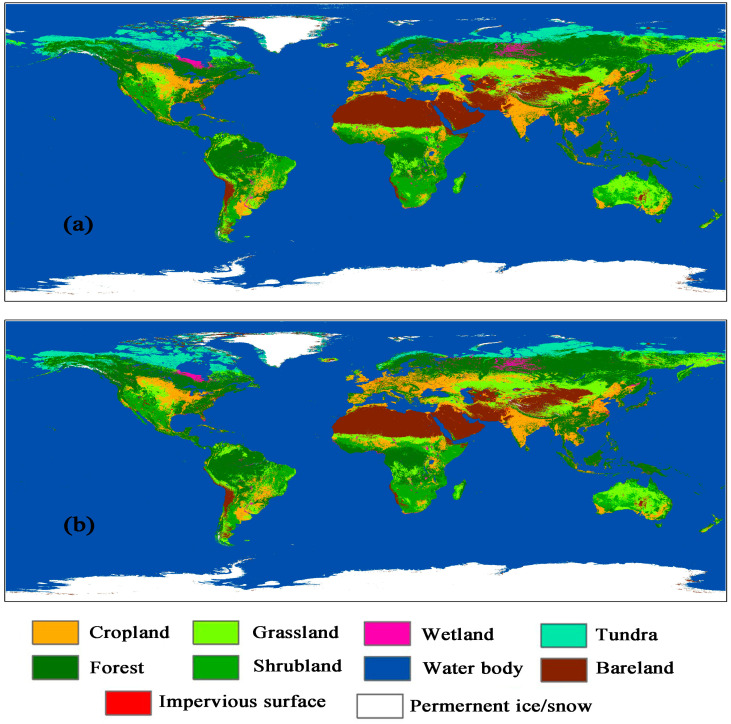
Global land cover maps in 2016 (**a**) and 2020 (**b**).

(1) Global geographical zoning. The global land cover types have regional characteristics affected by geographical location, topography, and climate. Dividing the world and training the model zone by zone can reduce the complexity of model parameters and improve classification accuracy. Here, the Koppen climate classification map [18] is used as the basis for geographical zoning.
(2) Training sample dataset preparation. The existing high-resolution global land cover products (FROM-GLC30 [19], GLC-FCS30 [20] and ESA WorldCover [21]) are used to create a sample dataset. Firstly, the resolution of the three products is unified to 10 m, and the classification system is also unified to the primary category with the same code. Secondly, extract the pixels with consistent code from the three products as initial training samples. Then, resample these samples to a resolution scale of 1 km based on the maximum proportion. Finally, select samples randomly and evenly according to the proportion of land cover types in each geographical zone.
(3) Surface Reflectance data pre-processing and model training. The surface reflectance product of MODIS, MOD09, is used as the basic data in the flow. Cloud pixel removal and median value composites are pre-processed to build global monthly time series surface reflectance maps. The land cover of 2020 (Figure 3b) is training using random forest model with sample dataset prepared in (2).
(4) Sample migration and model migration. Based on the land cover map of 2020, the initial classification results of land cover in 2016 is produced using the training model in (3). The model is re-trained using pixels with same encoding between land cover in 2016 (initial) and 2020. Then, the land cover of 2016 (final) (Figure 3a) is obtained using the new model.

The procedure has the advantages of high precision and high automation, which are specifically reflected in: (1) The global geographical zoning takes into account the differences and advantage types of each region, contributing to a high-precision model, even in the B&R region with complex terrain and different vegetation structure characteristics; (2) The strategy of building the sample set based on existing high-resolution products has increased the number and quality of samples; (3) The sample migration strategy improves the portability of the method, making it easier to realize the automatic production of land cover products for many years in succession.

Direct verification by selecting points on high-resolution images shows that the precision of land cover maps (Figure 3) produced by this flow is 88.97%, and the Kappa coefficient is 0.73. 

### 2.3. Setting Indicators to Assess Land Cover Condition and Change

In order to assess the spatial distribution and change trend of the main land cover types in the global land area and the “the Belt and Road” region from 2016 to 2020, 3 indicators are used: composition pattern, area change rate, and transformation trend of land cover types, which are shown in Table 1. 

## 3. Results

### 3.1. Land Cover Condition

**Figure 4:**
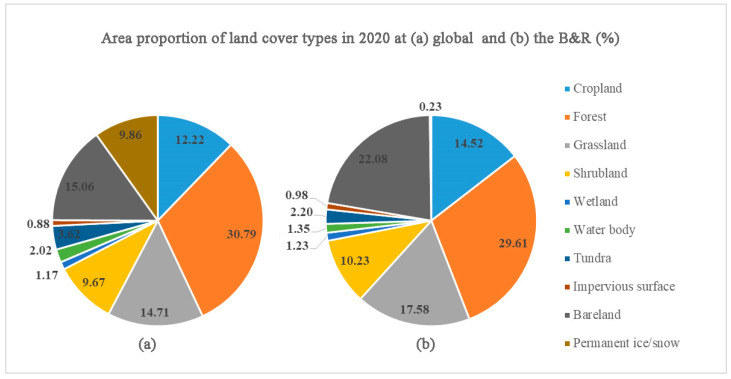
Area proportion of land cover types at global (**a**) and the B&R (**b**) scale in 2020.

The area proportion of land cover types in 2020 is shown in Table 2 and Figure 4, calculated at the global and B&R scale, respectively.

At the global scale, the area of cropland, forest, grassland, shrubland, wetland, water body, tundra, impervious surface, bareland, and permanent ice/snow are 17,954.35 × 10^3^ km^2^, 45,255.90 × 10^3^ km^2^, 21,617.28 × 10^3^ km^2^, 14,204.26 × 10^3^ km^2^, 1722.89 × 10^3^ km^2^, 2974.88 × 10^3^ km^2^, 5314.36 × 10^3^ km^2^, 1294.35 × 10^3^ km^2^, 22,131.90 × 10^3^ km^2^, and 14,489.22 × 10^3^ km^2^, accounting for 12.22%, 30.79%, 14.71%, 9.67%, 1.17%, 2.02%, 3.62%, 0.88%, 15.06%, and 9.86% globally, respectively. Among these types, forest accounts for the largest proportion, followed by bareland, grassland, and cropland, where impervious surface accounts for the smallest proportion.

At the B&R, except for water body, tundra, and permanent ice/snow, the areas of other types account for over 50% of their respective areas globally. Among these types, bareland, grassland, cropland, and impervious surface account for 92.28%, 75.22%, 74.78%, and 70.25% of their respective areas globally. Obviously, the B&R gathers most of the global vegetation resources and population resources. In the B&R, the proportions of forest, bareland, grassland, and cropland are 29.61%, 22.08%, 17.58% and 14.52%, respectively, accounting for 83.79% of the total area in B&R.

**Figure 5:**
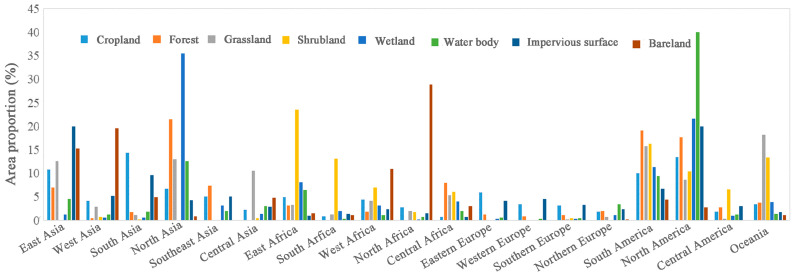
The distribution proportion of global land cover types (except tundra and permanent ice/snow) in various regions (except Antarctica) in 2020.

**Figure 6:**
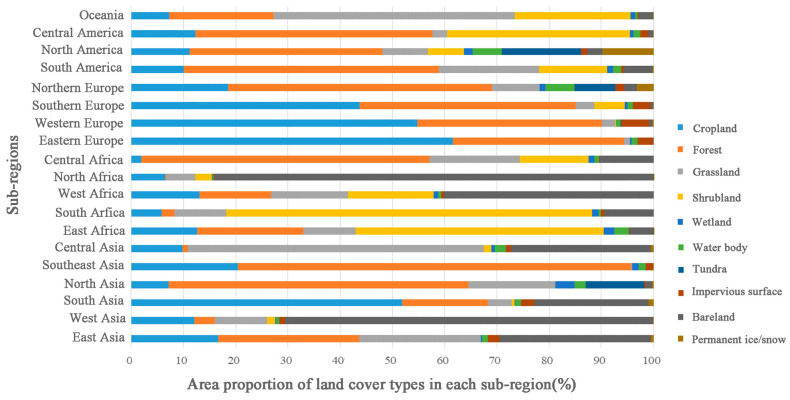
The area proportion of land cover types in each sub-region in 2020.

The spatial distribution of each type globally has distinct regional characteristics (Figure 5 and Figure 6). The cropland type is mainly distributed in South Asia, North America, East Asia, South America, and North Asia, accounting for 14.41%, 13.44%, 10.83%, 9.98%, and 6.77% of the total cropland area, respectively. The forest type land is mainly distributed in North Asia, South America, and North America, accounting for 21.52%, 19.12%, and 17.63% of the total forest area, respectively. The grassland type is mainly distributed in Oceania, South America, North Asia, and East Asia, accounting for 18.15%, 15.72%, 13.00%, and 12.58% of the total grassland area, respectively. The shrubland type is mainly distributed in East Africa, South America, Oceania, and South Africa, accounting for 23.52%, 16.31%, 13.35%, and 13.15% of the total shrub area, respectively. The wetland type is mainly distributed in North Asia, North America, and South America, accounting for 35.51%, 21.70%, and 11.32% of the total wetland area, respectively. The water body type is mainly distributed in North America and North Asia, accounting for 39.94% and 12.58% of the total water area, respectively. The tundra type is mainly distributed in North America and North Asia, accounting for 61.67% and 35.68% of the total tundra area, respectively. The bareland type is mainly distributed in North Africa, West Asia, East Asia, and West Africa, accounting for 28.91%, 19.56%, 15.26%, and 10.96% of the total area of bare land, respectively. The permanent ice/snow type is mainly distributed in Antarctica and North America, accounting for 83.50% and 14.61% of the total area, respectively.

### 3.2. Land Cover Change

**Figure 7:**
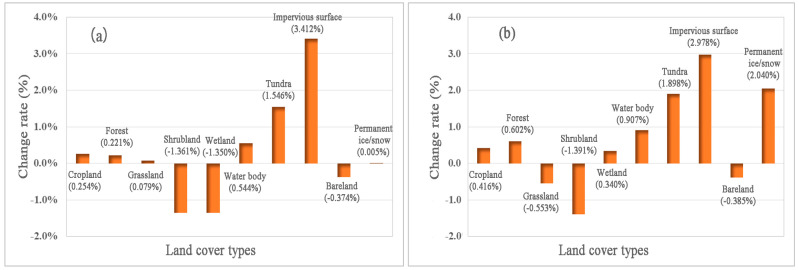
Change rate of land cover types from 2016 to 2020 globally (**a**) and at the B&R (**b**).

The net change and change rate for each type from 2016 to 2020 at global scale and the B&R are shown in Table 3 and Figure 7.

At the global scale, compared to 2016, the cropland, forest, grassland, water body, tundra, and impervious surface all increased in 2020, while shrubland, wetland, and bareland decreased. Among them, the impervious surface has the highest increase rate (3.41%), followed by tundra, with an increase rate of 1.55%. Cropland, forest, grassland, and water body have increased by 0.25%, 0.22%, 0.08% and 0.54%, respectively. Shrubland and wetland decreased significantly by 1.36% and 1.35%, respectively, while bareland decreased by 0.37%.

In the B&R area (Table 3 and Figure 7), compared with 2016, cropland, forest, wetland, water body, tundra, impervious surface, and permanent ice/snow cover are all increased in 2020, while the grassland, shrubland, and bareland are decreased. Among them, the growth rate of impervious surface is the highest at 2.98%, followed by permanent ice/snow and tundra, with growth rates of 2.04% and 1.90% respectively. Cropland, forest, and wetland increased by 0.42%, 0.60% and 0.34%, respectively, and waterbody increased by 0.91%. The shrubland decreased the most, by 1.39%, while the grassland and bareland decreased by 0.55% and 0.39%, respectively.

### 3.3. Mutual Transformation Characteristics between Various land Cover Types

The global land cover transition matrix from 2016 to 2020 is calculated and shown in Table 4. Among the cropland outflow in 2016, the area transferred to grassland is the largest, with 1058.32 × 10^3^ km^2^, followed by forest and shrubland, with 533.86 × 103 km^2^ and 495.04 × 103 km^2^, respectively; the area transferred into impervious surface is 152.06 × 103 km^2^. Among the forest outflow, the grassland transferred has the largest area, 845.26 × 10^3^ km^2^, followed by shrubland and cropland, with 547.54 × 10^3^ km^2^ and 508.56 × 10^3^ km^2^, respectively; the area transferred into impervious surface is relatively small, at 44.49 × 103 km ^2^. Among the grassland outflow, the area transferred into shrubland, cropland, and forest is relatively large, with 1024.71 × 10^3^ km^2^, 990.66 × 10^3^ km^2^ and 874.08 × 10^3^ km^2^, respectively; the area transferred into impervious surface is 60.93 × 10^3^ km^2^. Among the impervious surface outflow, the area transferred into cropland is the largest, with 125.22 × 10^3^ km^2^.

In the B&R (Table 5), the trend of land cover transition is consistent with the global scope. Among the cropland outflow in 2016, the area transferred to grassland is the largest, with 617.17 × 103 km ^2^, followed by forest and shrubland, with 400.18 × 103 km^2^ and 377.10 × 103 km^2^; the area transferred into an impervious surface is 113.22 × 103 km^2^. Among the outflow of forest, the area transferred to grassland is the largest, with 436.38 × 103 km ^2^, followed by cropland and shrubland, with 376.87 × 103 km ^2^ and 363.66 × 103 km ^2^; the area transferred into impervious surface is relatively small, at 19.72 × 103 km ^2^. Among the outflow of grassland, the areas transferred into shrubland and cropland are relatively large, with 693.47 × 103 km ^2^ and 590.75 × 103 km ^2^, respectively, followed by forest and bareland, with 483.65 × 103 km^2^ and 466.37 × 103 km ^2^, respectively; the area transferred into impervious surface is 38.58 × 103 km ^2^. Among the impervious surface outflow, the area transferred into cropland is the greatest, with 96.48 × 103 km ^2^.

## 4. Discussion

Human activities are the main factor in land cover change. Song et al. believed that of all land changes, 60% are associated with direct human activities and 40% with indirect drivers such as climate change [22]. According to the UN Population Fund (UNFPA) State of the World Population Report, the global population has increased from 7.2 billion in 2016 to 7.6 billion in 2020. The rapid growth of population requires more residential and facility land, leading to the expansion of impervious surface areas. Seto et al. reported a worldwide observed increase in urban land area of 58,000 km^2^ from 1970 to 2000 [23]. Liu et al. found that global urban extent has expanded by 9687 km^2^ per year from 1985 to 2015 [24]. Chen et al. found the global urban area increased from 625,000 to 1039,000 km^2^ during 2000–2012 [25]. Pan et al. found that from 2000 to 2005, the built-up area of 65 capital cities in the B&R increased from 23,696.25 to 29,257.51 km^2^, with an average growth rate of 370.75 km^2^ per year [26]. Wei et al. found the urban land occupation rate has increased from 0.32% in 2000 to 0.66% in 2018 (an increase of 250,600 km^2^) [27]. In our research, from 2016 to 2020, the impervious surface, closely related to urban land, in the global area and the B&R improved by 3.41% and 2.98% (Table 3), respectively. Among the new impervious surface, cropland contributes most with 49.68% (Table 4) in global and 57.67% (Table 5) in the B&R, followed by grassland and forest.

Furthermore, the change of cropland, forest, and grassland all directly related to human activities. In our research, the cropland increased 0.25% worldwide and 0.42% in the B&R, and the increased cropland manly comes from grassland, forest, and shrubland. The increase in population has led to an increase in demand for food. In order to increase the area of cropland to obtain more food, people have to convert natural land into cropland through reclamation in some countries [28,29,30]. Cao et al. found the cultivated land area has increased from 1.903 billion ha in 2000 to 1.960 billion ha in 2010 [31], and Chen et al. found the cropland in the B&R increased 3.73 × 104 km^2^ between 2000 and 2010 [32]. In fact, cropland changes exhibit significant disparities across different regions. In some developing countries, population growth and urbanization pressures have resulted in substantial decreases in cropland area, whereas certain developed countries may have experienced increases in cropland area due to advancements in agricultural technology and management practices.

International and national government policies play a certain driving role in land cover change. The B&R Initiative has promoted cross-border trade and investment and provided a broader market for agricultural products. In order to adapt to the growing demand, many countries have expanded their cultivated land to increase agricultural production and pursue economic benefits. Meanwhile, the infrastructure construction of the B&R initiative, including the improvement of roads, railways, and ports, has created favorable conditions for land cultivation and agricultural product transportation, and further promoted the change of cropland use [32]. Moreover, policies formulated by some countries for socio-economic development can also affect changes in land cover. For example, since 1980s, the Chinese government has implemented National Afforestation Project and achieved remarkable results [33]. China has the largest afforestation and reforestation area globally, with annual afforestation rates ranking among the highest in the world. As of 2019, China’s forest coverage had surpassed 23%, playing an active role in protecting ecological balance and biodiversity. In this paper, the forest is indicated as increasing by 0.22% in global and 0.60% in the B&R, which is contrary to the prevailing view that forest area has declined, but consistent with Song that tree cover has increased by 7.1% from 1982 to 2016 [22].

Natural environmental factors such as climate can also affect the state and trend of land cover changes. Climate change has led to an increase in extreme weather events, such as frequent droughts, floods, and rainstorm. These extreme weather conditions directly affect soil moisture and water circulation, and have a significant impact on sustainable land use and vegetation growth [34,35,36]. In addition, climate change has also had an impact on biodiversity and ecosystems, thereby affecting the stability of land cover. Climate change may lead to the migration of some species and changes in habitats, thereby altering vegetation types and distribution. For example, under high-temperature and dry climate conditions, plants that were originally adapted to humid environments may disappear, thereby altering the vegetation cover on the surface [37].

## 5. Conclusions

This paper innovatively proposed a procedure to produce low-resolution land cover maps with good continuous consistency and high accuracy. This procedure reduces the impact of regional differences on accuracy through geographical zoning in large-scale land cover mapping. The idea of using several published high-resolution land cover classification products makes the acquisition of initial samples more efficient, easy, and cost-effective. In addition, sample migration and model migration makes it more automated to produce continuous land cover maps for many years.

In addition, this paper has carried out an assessment of land cover condition and change at global scale and the B&R region, including the spatial distribution of land cover, the change of land cover and the mutual transformation characteristics among various types. Based on the classification maps of 2016 and 2020, the land cover condition, land cover change, and mutual transformation characteristics between various types at global scale and B&R region are assessed, and the following conclusions can be drawn:

(1) Globally, the cropland, forest, grassland, shrub, wetland, water body, tundra, impervious surface, bareland, and permanent ice/snow cover in 2020 account for 12.22%, 30.79%, 14.71%, 9.67%, 1.17%, 2.02%, 3.62%, 0.88%, 15.06%, and 9.86% of the total land area, respectively. In terms of spatial distribution, the cropland is mainly distributed in South Asia, North America, East Asia, South America, and North Asia; forest is mainly distributed in North Asia, South America, and North America; grassland is mainly distributed in Oceania, South America, North Asia, and East Asia; shrubland is mainly distributed in East Africa, South America, Oceania, and South Africa; wetland is mainly distributed in North Asia, North America, and South America; waterbody is mainly distributed in North America and North Asia; tundra is mainly distributed in North America and North Asia; bareland is mainly distributed in North Africa, West Asia, East Asia, and West Africa; and permanent ice/snow is mainly distributed in Antarctica and North America. Compared to 2016, in 2020, cropland, forest, grassland, waterbody, tundra, and impervious surface increased, while shrubland, wetland, and bareland decreased. Impervious surface has the highest growth rate, followed by tundra, while shrubland and wetland decreased significantly. Among the cropland outflow in 2016, the area transferred to grassland is the largest, followed by forest and shrubland. Among the outflow of forest, the area transferred to grassland is the largest, followed by shrubland and cropland, while the area transferred to impervious surface is relatively small. Among the outflow of grassland, the top three in terms of area are shrubland, cropland, and forest. Among the outflow of impervious surface, the area transferred to cropland is the largest.
(2) In the B&R region, cropland, forest, grassland, shrubland, wetland, water body, tundra, impervious surface, bareland, and permanent ice/snow respectively account for 14.52%, 29.61%, 17.58%, 10.23%, 1.23%, 1.35%, 2.20%, 0.98%, 22.08% and 0.23% of the total area of the B&R, accounting for 74.78%, 60.52%, 75.22%, 66.60%, 65.89%, 41.93%, 38.22%, 70.25%, 92.28% and 1.44% of the total area of the same type globally. Compared to 2016, in 2020, there was an increase in cropland, forest, wetland, water body, tundra, impervious surface, and permanent ice/snow, while grassland, shrubland, and bareland decreased. Among them, the increase rate of impervious surface is the highest, followed by permanent ice/snow and tundra, while shrubland has the highest decrease rate. The types of land cover outflow from 2016 and inflow to 2020 are basically consistent with those of the global area. Among the cropland outflow in 2016, the area of grassland is the largest, followed by forest and shrubland. Among the forest outflow, the area transferred into grassland is the largest, followed by cropland and shrubland. Among the grassland outflow, the area transferred into shrubland and cropland is greater, followed by forest and bareland. Among the impervious surfaces outflow, the area transferred into cropland is the greatest.

The procedure proposed in this paper solved the problem of discontinuity and low accuracy in current low-resolution land cover classification products. This procedure is conductive to achieving land cover monitoring for consecutive years globally and in the B&R, which is of great significance to reflect the advantages and achievements of the B&R initiative and promote the major findings of the B&R study.

The assessment results in this paper will help to clarify the spatial pattern of land cover change in the five years from 2016 to 2020, so as to provide valuable scientific information for the global realization of SDGs and the construction of the B&R.

## Figures and Tables

**Table 1:** List of evaluation indicators for land cover status and change.

Evaluation Content	Indicators	Indicator Meaning	Formula *
Land cover condition	Composition pattern of land cover types	The area proportion of every land cover type calculated by the land cover map of 2020.	$P_{i}=\frac{S_{i}}{S}*100\%$
Land cover change	Area change rate of land cover types	The area change of all land cover types from 2016 to 2020.	$E_{i}=\frac{S_{i,2020}-S_{i,2016}}{S_{i,2016}}*100\%$
Mutual transformation characteristics between various land cover types	Transformation trend of land cover types	The transition matrix of land cover types between 2016 and 2020.	$\left\{\begin{array}{c} {PA}_{ab}=\frac{S_{ab}}{\sum_{b=1}^{n}S_{ab}}*100\%\\ {PB}_{ab}=\frac{S_{ab}}{\sum_{b=1}^{n}S_{ab}}*100\% \end{array}\right.$

*: $P_{i}$ is the area proportion of the *i* type of land cover map of 2020; $S_{i}$ is the area of the *i* type of land cover map of 2020; S is the total area; $E_{i}$ is the area change rate of the *i* type during the evaluation period; $S_{i,2016}$ is the area of *i* type at 2016; $S_{i,2020}$ is the area of *i* type at 2020; *a* is the land cover type of 2016, *b* is the land cover type of 2020, $S_{ab}$ is the area of land cover type, ${PA}_{ab}$ is the proportion of the transformation from the *a* type in 2016 to the b type in 2020. ${PB}_{ab}$ is the proportion of *b* type in 2020 transformed from *a* type in 2016.

**Table 2:** Composition of global and the B&R land cover types in 2020.

Range	Global		B&R		
Types	Area (10^3^ km^2^)	Proportion ^1^ (%)	Area (10^3^ km^2^)	Proportion ^2^ (%)	Proportion ^3^ (%)
Cropland	17,954.35	12.22	13,425.70	74.78	14.52
Forest	45,255.90	30.79	27,388.19	60.52	29.61
Grassland	21,617.28	14.71	16,260.04	75.22	17.58
Shrubland	14,204.26	9.67	9459.67	66.60	10.23
Wetland	1722.89	1.17	1135.25	65.89	1.23
Water body	2974.88	2.02	1247.52	41.93	1.35
Tundra	5314.36	3.62	2031.02	38.22	2.20
Impervious surface	1294.35	0.88	909.23	70.25	0.98
Bareland	22,131.90	15.06	20,423.38	92.28	22.08
Permanent ice/snow	14,489.22	9.86	208.86	1.44	0.23

^1^ Area proportion of a type in total global land area. The calculation formula is $S_{i}/S_{g}$, where $S_{i}$ means area of *i* type, $S_{g}$ means area of the total global land. ^2^ Area proportion of a type in global land area of that type. The calculation formula is $S_{i,\;B\&R}/S_{i,\;global}$, where $S_{i,\;B\&R}$ means area of *i* type in B&R, $S_{i,\;global}$ means area of *i* type in global. ^3^ Area proportion of a type in B&R land area. The calculation formula is $S_{i}/S_{B\&R}$, where $S_{i}$ means area of *i* type, $S_{B\&R}$ means area of the B&R land area.

**Table 3:** The net change and change rate for each type from 2016 to 2020.

Types	Global				The B&R			
	2016	2020	Net Change	Rate of Change	2016	2020	Net Change	Rate of Change
Cropland	17,908.789	17,954.352	45.563	0.25	13,370.07	13,425.70	55.63	0.42
Forest	45,156.080	45,255.897	99.817	0.22	27,224.38	27,388.19	163.81	0.60
Grassland	21,600.303	21,617.276	16.973	0.08	16,350.39	16,260.04	−90.35	−0.55
Shrubland	14,400.186	14,204.264	−195.922	−1.36	9593.13	9459.67	−133.46	−1.39
Wetland	1746.457	1722.887	−23.57	−1.35	1131.41	1135.25	3.84	0.34
Water body	2958.772	2974.881	16.109	0.54	1236.30	1247.52	11.22	0.91
Tundra	5233.475	5314.363	80.888	1.55	1993.18	2031.02	37.84	1.90
Impervious surface	1251.652	1294.354	42.702	3.41	882.94	909.23	26.30	2.98
Bareland	22,214.921	22,131.899	−83.022	−0.37	20,502.37	20,423.38	−78.99	−0.39
Permanent ice/snow	14,488.508	14,489.215	0.707	0.01	204.69	208.86	4.18	2.04

**Table 4:** Transition matrix between 2016 and 2020 globally (unit: 10^3^ km^2^).

	2020	Cropland	Forest	Grassland	Shrubland	Wetland	Water Body	Tundra	Impervious Surface	Bareland	Permanent Ice/Snow	Total for 2016	Total Outflow
2016													
Cropland		15,546.75	533.86	1058.32	495.04	33.07	12.59	1.74	152.06	75.34	0.02	17,908.79	2362.04
Forest		508.56	42,866.20	845.26	547.54	182.97	83.64	67.60	44.49	9.29	0.57	45,156.11	2289.90
Grassland		990.66	874.08	17,786.16	1024.71	156.37	22.65	167.87	60.93	516.00	0.88	21,600.30	3814.15
Shrubland		619.24	585.24	1062.78	11,879.50	46.73	11.54	0.73	26.01	168.39	0.02	14,400.18	2520.68
Wetland		32.64	205.92	154.98	41.51	1238.88	41.78	16.55	5.65	8.55	0.01	1746.46	507.58
Water body		8.10	80.72	19.97	9.52	38.23	2627.69	69.34	6.83	43.71	54.67	2958.77	331.08
Tundra		1.42	66.37	120.96	0.48	14.40	50.36	4884.48	0.04	90.43	4.55	5233.48	349.00
Impervious surface		125.22	36.60	50.77	28.47	4.76	10.31	0.15	988.25	7.02	0.10	1251.65	263.40
Bareland		121.77	5.74	517.42	177.49	7.45	57.60	101.86	10.06	21,157.92	57.61	22,214.92	1057.01
Permanent ice/snow		0.00	1.16	0.65	0.01	0.04	56.72	4.05	0.03	55.26	14,370.80	14,488.71	117.91
Total for 2020		17,954.35	45,255.88	21,617.28	14,204.26	1722.89	2974.88	5314.36	1294.35	22,131.90	14,489.22	——	——
Total inflow		2407.61	2389.68	3831.12	2324.76	484.01	347.19	429.88	306.10	973.98	118.42	——	——

**Table 5:** Transition matrix between 2016 and 2020 in B&R (unit: 10^3^ km^2^).

	2020	Cropland	Forest	Grassland	Shrubland	Wetland	Water Body	Tundra	Impervious Surface	Bareland	Permanent Ice/Snow	Total for 2016	Total Outflow
2016													
Cropland		11,744.94	400.18	617.17	377.10	30.65	11.51	0.00	113.22	75.28	0.02	13,370.07	1625.13
Forest		376.87	25,875.84	436.38	363.66	105.56	25.99	19.25	19.72	1.02	0.11	27,224.38	1348.55
Grassland		590.75	483.65	13,858.53	693.47	92.09	10.83	115.40	38.58	466.37	0.72	16,350.39	2491.86
Shrubland		461.48	429.60	685.09	7873.96	35.24	0.80	0.33	5.49	101.13	0.00	9593.13	1719.16
Wetland		27.27	125.02	86.28	26.21	828.23	16.61	8.41	5.05	8.32	0.00	1131.41	303.17
Water body		6.83	26.44	6.51	0.53	19.97	1121.50	25.23	5.22	23.31	0.76	1236.30	114.80
Tundra		0.00	29.07	73.78	0.06	12.00	18.23	1843.09	0.01	14.77	2.16	1993.19	150.09
Impervious surface		96.48	17.67	30.39	6.70	4.33	8.21	0.07	712.91	6.06	0.10	882.94	170.02
Bareland		121.08	0.71	465.54	117.97	7.17	32.60	17.28	9.01	19,708.72	22.30	20,502.37	793.65
Permanent ice/snow		0.00	0.01	0.36	0.00	0.00	1.24	1.97	0.03	18.39	182.68	204.69	22.00
Total for 2020		13,425.70	27,388.19	16,260.04	9459.67	1135.25	1247.52	2031.02	909.23	20,423.38	208.86	——	——
Total inflow		1680.76	1512.35	2401.51	1585.70	307.02	126.02	187.93	196.32	714.66	26.18	——	——

## Data Availability

Not applicable.
